# Supplementary material for: The Systems Biology Research Tool: evolvable open-source software
Source: BMC Syst Biol. 2008 Jun 29;2:55. doi: 10.1186/1752-0509-2-55 (PMC2446383; doi:10.1186/1752-0509-2-55)
Supplement: Additional file 1 — SBRT Archive. An archive of the current version of the Systems Biology Research Tool. [file 1752-0509-2-55-S1.zip › sbrt-1.4.0/doc/developers_guide/api/sbrt/shell/text/math/LinCombTermFormatV1.html]

LinCombTermFormatV1


|  |  |  |
| --- | --- | --- |
| **Overview** \| **Package** \| **Class** \| **Use** \| **Tree** \| **Deprecated** \| **Index** \| **Help** | |  |
| **PREV CLASS**   **NEXT CLASS** | **FRAMES**    **NO FRAMES**     **All Classes** |  |
| SUMMARY: NESTED \| FIELD \| CONSTR \| METHOD | DETAIL: FIELD \| CONSTR \| METHOD |  |


---


## sbrt.shell.text.math Class LinCombTermFormatV1<V>

```
java.lang.Object
  sbrt.shell.text.math.LinCombTermFormatV1<V>
```

**Type Parameters:**: `V` - the variable type.

**All Implemented Interfaces:**: Format, Formatter<LinearComb.Term<? extends V>>, LinearCombTermFormat<V>, Parser<LinearComb.Term<? extends V>>, SimpleFormat<LinearComb.Term<? extends V>>

---

``` public final class LinCombTermFormatV1<V> extends java.lang.Object implements LinearCombTermFormat<V> ```

This class is a concrete implementation of `LinearCombTermFormat`.
Terms have the following format: `coefficient variable`.

**Author:**
:   This class was written and documented by
    Jeremiah Wright while in the Wagner lab.

---

**Constructor Summary**

`LinCombTermFormatV1(SimpleFormat<V> variableFormat)`
  
          Constructs a new linear combination term format using
the provided formats.


| **Method Summary** | |
| --- | --- |
| `java.lang.String` | `format(LinearComb.Term<? extends V> term)`             Returns a formatted string representation of the provided term. |
| `CoeffFormatV1` | `getCoeffFormat()`             Returns the format used for coefficients. |
| `SimpleFormat<V>` | `getVariableFormat()`             Returns the format used for variables. |
| `LinearComb.Term<V>` | `parse(java.lang.String term)`             Parses the provided string and returns its corresponding term. |
| `LinearComb.Term<V>` | `parse(java.lang.String coefficient, java.lang.String variable)`             Parses the provided strings and returns their corresponding term. |

**Methods inherited from class java.lang.Object**

`clone, equals, finalize, getClass, hashCode, notify, notifyAll, toString, wait, wait, wait`

**Methods inherited from interface sbrt.shell.text.Formatter**

`format`

**Constructor Detail**

### LinCombTermFormatV1

```
public LinCombTermFormatV1(SimpleFormat<V> variableFormat)
```

:   Constructs a new linear combination term format using
    the provided formats.

    **Parameters:**: `variableFormat` - the format to use for variables.


**Method Detail**

### getVariableFormat

```
public SimpleFormat<V> getVariableFormat()
```

:   Returns the format used for variables.

    :   **Returns:**: the format used for variables.

---


### getCoeffFormat

```
public CoeffFormatV1 getCoeffFormat()
```

:   Returns the format used for coefficients.

    :   **Returns:**: the format used for coefficients.

---


### format

```
public java.lang.String format(LinearComb.Term<? extends V> term)
```

:   Returns a formatted string representation of the
    provided term.

    :   **Specified by:**: `format` in interface `LinearCombTermFormat<V>`
    :   **Parameters:**: `term` - the term to be formatted. **Returns:**: a formatted string representation of the provided term.

---


### parse

```
public LinearComb.Term<V> parse(java.lang.String term)
```

:   Parses the provided string and returns its
    corresponding term.

    :   **Specified by:**: `parse` in interface `LinearCombTermFormat<V>` **Specified by:**: `parse` in interface `Parser<LinearComb.Term<? extends V>>`
    :   **Parameters:**: `term` - the string to be parsed. **Returns:**: the term corresponding to the provided string.

---


### parse

```
public LinearComb.Term<V> parse(java.lang.String coefficient,
                                java.lang.String variable)
```

:   Parses the provided strings and returns their
    corresponding term.

    :   **Parameters:**: `coefficient` - the string of the coefficient to be parsed.: `variable` - the string of the variable to be parsed. **Returns:**: the term corresponding to the provided strings.


---


|  |  |  |
| --- | --- | --- |
| **Overview** \| **Package** \| **Class** \| **Use** \| **Tree** \| **Deprecated** \| **Index** \| **Help** | |  |
| **PREV CLASS**   **NEXT CLASS** | **FRAMES**    **NO FRAMES**     **All Classes** |  |
| SUMMARY: NESTED \| FIELD \| CONSTR \| METHOD | DETAIL: FIELD \| CONSTR \| METHOD |  |


---
